# Portable Low‐Field Magnetic Resonance Imaging in People With Human Immunodeficiency Virus

**DOI:** 10.1002/acn3.70237

**Published:** 2025-11-06

**Authors:** Annabel Sorby‐Adams, Malachi Keo, Jennifer Guo, Daire Daly, Richard Ahern, Kimon Zachary, Gregory Robbins, Rajesh T. Gandhi, Bragi Sveinsson, Adam de Havenon, Kevin N. Sheth, Otto Rapalino, Juan Eugenio Iglesias Gonzales, W. Taylor Kimberly, Shibani S. Mukerji

**Affiliations:** ^1^ Department of Neurology Massachusetts General Hospital and Harvard Medical School Boston Massachusetts USA; ^2^ Center for Genomic Medicine Massachusetts General Hospital and Harvard Medical School Boston Massachusetts USA; ^3^ Division of Infectious Diseases Massachusetts General Hospital and Harvard Medical School Boston Massachusetts USA; ^4^ Athinoula A. Martinos Center for Biomedical Imaging, Department of Radiology Massachusetts General Hospital and Harvard Medical School Boston Massachusetts USA; ^5^ Department of Neurology, Center for Brain & Mind Health Yale New Haven Hospital and Yale School of Medicine New Haven Connecticut USA; ^6^ Division of Neuroradiology, Department of Radiology Massachusetts General Hospital and Harvard Medical School Boston Massachusetts USA; ^7^ Centre for Medical Image Computing University College London London UK; ^8^ Computer Science and Artificial Intelligence Laboratory Massachusetts Institute of Technology Cambridge Massachusetts USA; ^9^ Vaccine and Immunotherapy Center Massachusetts General Hospital Boston Massachusetts USA

**Keywords:** aging, artificial intelligence, atrophy, HIV, low‐field MRI, white matter hyperintensities

## Abstract

**Objective:**

The aging population of people with HIV (PWH) raises heightened concerns regarding accelerated aging and dementia. Portable, low‐field MRI (LF‐MRI) is an innovative technology that could enhance access and facilitate routine monitoring of PWH. We sought to evaluate the feasibility of LF‐MRI and apply a machine learning (ML) segmentation algorithm to examine atrophy and white matter hyperintensities (WMH) in PWH compared to people without HIV (PWoH) of similar age.

**Methods:**

Individuals with a confirmed diagnosis of HIV on antiretroviral therapy underwent LF‐MRI (64 mT) acquisition in the outpatient neurology clinic. PWoH with > 1 vascular comorbidity (VC cohort, *n* = 25) or with mild cognitive impairment (MCI cohort, *n* = 24) due to Alzheimer's disease served as comparators. LF‐MRI brain region segmentations were derived using the ML algorithm WMH‐SynthSeg in FreeSurfer. Brain regions corrected for intracranial volume were compared between cohorts after adjusting for age and sex.

**Results:**

Thirty virally suppressed PWH were included. LF‐MRI derived brain volumes from PWH demonstrated a reduction in volume of the caudate relative to PWoH with VC (*p* < 0.05). Volume of the putamen and white matter was reduced in PWH compared to VC (*p* < 0.05). Hippocampal volume was comparable between PWH and PWoH (*p* ≥ 0.05), while volume of the amygdala was reduced in those with MCI alone (*p* < 0.05). No differences in WMH were seen between these cohorts (*p* > 0.05).

**Interpretation:**

LF‐MRI is feasible in an outpatient setting, and ML algorithms enable detection of regional atrophy and WMH in PWH. LF‐MRI may enable more frequent monitoring and earlier detection of atrophy in at‐risk populations.

## 1 Introduction

Recent estimates suggest that 54% of the United States (US) population with human immunodeficiency virus (HIV) is aged 50 years or older [1], necessitating a shift toward treatment and care for an aging population with HIV [2]. Model‐based projections suggest that 16%–22% of 60‐year‐olds with HIV may develop premature age‐associated dementia, compared to 13%–15% in the general population [3, 4, 5]. As concerns about brain health rise and increasing emphasis on neurological assessments in US and global guidelines, clinicians caring for people with HIV (PWH) are prioritizing neurology‐related diagnosis and management [6, 7, 8].

High‐field (HF) magnetic resonance imaging (MRI), operating at > 1 Tesla (T), is the gold standard for assessing age‐related changes in the brain [9], including vascular pathology (e.g., cerebral small vessel disease and associated white matter hyperintensities (WMH) [10]), neurodegenerative (e.g., hippocampal atrophy [11]), and HIV‐related structural alterations (e.g., subcortical atrophy). However, high cost, limited availability, and stringent operating requirements render HF‐MRI access challenging for many people. For the approximately 60% of PWH residing in sub‐Saharan Africa and resource‐constrained regions, routine screening with HF‐MRI is not feasible [12]. Even in US locations where HF‐MRI is available, PWH encounter financial and administrative barriers, including transportation challenges and the need for work flexibility to accommodate imaging appointments outside of routine check‐ups [13, 14].

Recent advances in MRI technology have allowed for the development of portable, low‐field (LF) MRI systems operating at magnetic field strengths below 1 T [15]. These systems are more compact, operate without liquid cryogens, and are 4–12 times less expensive than HF‐MRI counterparts [16, 17, 18, 19]. They can be placed in clinical settings without magnetic shielding, making them an attractive alternative to HF‐MRI, especially in clinics where there are no feasible options for neuroimaging. For research applications, publicly available machine learning (ML) algorithms have been validated to overcome the low signal‐to‐noise ratio challenges inherent in LF‐MRI brain scans and enable automatic segmentation for quantitative morphometry [20].

Despite antiretroviral therapy (ART), PWH exhibit unique patterns of brain atrophy, characterized by subcortical atrophy, particularly in the caudate nucleus, and a higher prevalence of WMH compared to people without HIV (PWoH) [21, 22]. These findings suggest appreciable MRI biomarkers such as regional atrophy, which is associated with HIV duration and CD4+ T‐cell counts, and increased WMH, which may reflect both HIV characteristics and underlying cardiovascular risk factors [21, 22, 23]. To assess these interrelated neurological changes, we conducted a study of LF‐MRI in PWH on ART from outpatient Infectious Disease clinics. Our aim was to evaluate the feasibility of LF‐MRI and determine the accuracy and utility of ML‐derived brain volumes in distinguishing between PWH and PWoH of similar age with vascular comorbidities or mild cognitive impairment (MCI) associated with Alzheimer's disease (AD).

## 2 Materials and Methods

### 2.1 Study Design

This was a single‐center, prospective, observational study performed at an academic tertiary hospital in the US (Massachusetts General Hospital; MGH). English‐speaking individuals with a confirmed diagnosis of HIV on ART were recruited from the Infectious Diseases clinic between January and June 2024. The presence of neurological symptoms or signs was not required for enrollment. Recruitment aimed to gather a representative population of PWH in Suffolk County, Massachusetts, based on 2021 prevalence rate data [24]. Exclusion criteria were age < 18 years, pregnancy, electrically stimulated implants such as cardiac pacemakers, and a body weight exceeding 400 lbs (181.4 kg). All imaging was acquired for research purposes under an Institutional Review Board (IRB) approved protocol (2023P002808) with informed consent obtained prospectively from participants.

### 2.2 Image Acquisition and Clinical Assessments

A clinically approved, LF‐MRI system operating at a field strength of 64 mT was used for all imaging (Hyperfine Inc., hardware v1.9, software v8.7.0) [25]. The scanner was located in the outpatient neurology clinic to facilitate imaging at the point of care. The imaging protocol for all participants included *T*_1_‐weighted, *T*_2_‐weighted, and *T*_2_ fluid‐attenuated inversion recovery (FLAIR) sequences acquired over 27 min, with DICOM images automatically uploaded to a cloud‐based platform (Purview Image) following acquisition (full imaging parameters reported in Table S1). On the day of imaging, medical history, blood pressure, blood samples for T‐cell counts and HIV‐1 viral load, and the Montreal Cognitive Assessment (MoCA) battery were collected from all participants. ACC/AHA 10‐Year Atherosclerotic Cardiovascular Disease (ASCVD) risk [26] was calculated based on age, sex, race, blood pressure, cholesterol levels, history of diabetes, smoking status, and antihypertensive use.

Retrospective analysis of prospectively acquired LF‐MRI images in PWoH was utilized for comparison. These included scans from PWoH with at least 1 vascular comorbidity (VC) and probable vascular cognitive impairment presenting to the Yale New Haven Hospital between December 2021 and July 2022 [27]. A second cohort comprised scans from PWoH presenting to the MGH Memory Disorders clinic between February 2023 and August 2024 with a diagnosis of MCI due to suspected AD [25]. Demographics and medical history were collected for both cohorts, including MoCA scores where available, with consent obtained prospectively from the individual or their legally authorized representative under IRB approved protocol.

### 2.3 Image Postprocessing

Raw LF‐MRI images from each cohort were manually inspected for quality prior to conversion into Neuroimaging Informatics Technology Initiative (NIfTI) open file format. LF axial *T*_2_ FLAIR NIfTI images from all participants were processed centrally through FreeSurfer (v7.3.2) ML algorithm WMH‐SynthSeg in accordance with ENIGMA consortium standards [20, 28, 29]. WMH‐SynthSeg produces a super‐resolved, 1 mm isotropic segmentation of the brain comprising 32 anatomical regions of interest whilst simultaneously segmenting areas of periventricular WMH. Bilateral hemispheric segmentation volumes (e.g., left and right hippocampi) were summed and averaged. Regions were then grouped as follows: global (white matter and cortex), ventricular (lateral, third and fourth ventricles), diencephalic‐thalamic complex (DTC; thalamus, ventral diencephalon (DC), and nucleus accumbens), basal ganglia (pallidum, putamen and caudate), medial temporal (amygdala and hippocampus), and WMH. To enable comparison between the VC, MCI, and PWH cohorts, volumes were corrected for total intracranial volume (ICV) to account for differences in head size (region divided by total ICV), except for WMH volume.

### 2.4 Postprocessing Validation

To validate the accuracy of LF‐MRI derived segmentations in PWH, available HF‐MRI (1.5–3 T) acquired for clinical care or research purposes within 1 year of LF acquisition was used for comparison to LF‐MRI counterparts. *T*_2_ FLAIR HF images were processed through WMH‐SynthSeg and structures grouped as described. To enable assessment of spatial overlap between regions using the Dice similarity coefficient, HF images were co‐registered to LF counterparts using a nonlinear approach implemented in NiftyReg [30] and computed as previously described [25]. The absolute symmetrized percent difference (ASPD) between HF and LF volumes was also calculated as an additional measure of agreement between field strengths [25]. A Dice of 1 and ASPD of 0 indicated perfect agreement in spatial overlap and volume between LF and HF counterparts, respectively. To contextualize ML derived WMH volumes with visual assessment of WMH burden, LF‐MRI images were graded by a trained neurologist using the Fazekas scale, a standardized assessment for small vessel disease burden and used in clinical decision‐making pathways [22, 31, 32, 33] on an ordinal scale of 0–3 for both the periventricular and deep white matter.

### 2.5 Statistical Analysis

Data were analyzed using STATA (v18.0) and R (v2024.09.0). ASCVD risk was calculated using the ASCVD module in STATA [34]. LF and HF derived volumes were tested for normality using the Shapiro–Wilk test, where no significant deviations from normality were detected (all *p* > 0.05). Demographic differences between cohorts were analyzed via Wilcoxon rank sum exact tests. Pearson's correlation coefficients (*r*) were performed to assess the association between brain volumes derived from HF and LF counterparts in PWH and to evaluate associations between brain volumes and ASCVD risk and cognitive performance on the MoCA. Spearman's correlations (*r*) were performed to assess the association between LF derived WMH volumes and the visually graded Fazekas scale. Bootstrap estimates with 500 random samples were used to calculate 95% confidence intervals (CIs). Comparisons between three or more groups were evaluated using Kruskal‐Wallis tests with Dunn's post hoc. Comparisons between two groups were analyzed via Wilcoxon rank sum exact tests. Multivariate linear regression analysis was performed to determine the relationship between brain volumes of interest and HIV characteristics when adjusted for age and sex (with CD4+ T‐cell count, CD4+ T‐cell nadir, HIV‐1 viral load, and ART duration as predictors). Multivariate linear regression analysis was also performed to determine the relationship between brain volumes of interest and cohort (PWH, VC, MCI) when adjusted for age and sex, with PWH as the reference cohort. Pairwise comparisons of adjusted marginal mean with Bonferroni correction were reported as adjusted effect size and multiplicity‐adjusted *p* values. Pearson's and Spearman's correlations were defined as strong (*r* > 0.7), moderate (*r* ≤ 0.7 to < 0.5), fair (*r* ≤ 0.5 to ≤ 0.3), or poor (*r* < 0.3). Data are reported as median, 95% CI, and interquartile range (IQR). A *p* value < 0.05 was considered statistically significant.

## 3 Results

### 3.1 Cohort Demographics

Thirty‐four PWH on ART were enrolled and completed all study procedures in the same clinic room within 90 min of providing consent. Of the 34 participants, four were excluded due to incomplete capture of the temporal lobes (*n* = 3) or had prior excision of a tumor resulting in WMH pathology considered unrelated to their HIV diagnosis (*n* = 1). The final cohort included *n* = 30 PWH with a median HIV infection duration of 27 years, most of whom were on stable long‐term ART. Forty‐nine PWoH were included for comparison, including *n* = 25 individuals with at least 1 vascular risk factor and probable vascular cognitive impairment (VC), and *n* = 24 individuals with MCI due to AD. Demographics of each cohort are reported in Table 1.

**TABLE 1:** Cohort demographics. People with HIV (PWH), people without HIV (PWoH) with > 1 vascular comorbidity (vascular comorbidities cohort; VC) or mild cognitive impairment (MCI) due to suspected Alzheimer's disease.

	PWH	VC	MCI
	*N* = 30	*N* = 25	*N* = 24
Age, median [IQR], years	62 [59–67]	63 [58–68]	72 [68–78]
Sex, *n* (%)			
Female	10 (33)	12 (48)	11 (46)
Male	20 (67)	13 (52)	13 (54)
Race, *n* (%)			
Black or African American	12 (40)	8 (32)	0 (0)
White	15 (50)	14 (56)	24 (100)
Other	3 (10)	3 (12)	0 (0)
Ethnic group in North America, *n* (%)			
Hispanic	8 (27)	4 (16)	0 (0)
Non‐Hispanic	22 (73)	21 (84)	24 (100)
Cardiovascular risk factors, *n* (%)			
Hypertension	19 (63)	19 (76)	7 (30)
Hyperlipidemia	18 (60)	19 (76)	9 (38)
Atrial fibrillation	1 (3)	5 (20)	0 (0)
Diabetes	7 (23)	11 (44)	1 (4)
Cumulative risk, median [IQR]	2 [1–2]	2 [1–3]	1 [0–1]
Montreal cognitive assessment (MoCA)			
MoCA available, *n* (%)	29 (97)	16 (64)	20 (83)
Median score [IQR]	23 [20–25]	23 [19–25]	22 [20–26]
Score distribution, *n* (%)			
Normal cognitive performance (≥ 26)	6 (20)	4 (16)	6 (25)
Mild impairment (18–25)	20 (67)	10 (40)	14 (58)
Moderate impairment (10–17)	3 (10)	2 (8)	0 (0)
HIV characteristics			
HIV infection duration, median [IQR], years	27 [23–35]		
CD4^+^ T‐cell count, *n* (%)			
< 500 cells/mm^3^	4 (13)		
≥ 500 cells/mm^3^	26 (87)		
CD4^+^ T‐cell nadir, *n* (%)^a^			
< 200 cells/mm^3^	9 (30)		
200–349 cells/mm^3^	4 (13)		
> 350 cells/mm^3^	6 (20)		
Antiretroviral therapy (ART) characteristics			
ART duration, median [IQR], years	24 [16–29]		
HIV‐1 viral load, *n* (%)^b^			
Virally suppressed (< 200 copies/mL)	30 (100)		
Virologic suppression duration, *n* (%)			
≥ 1 year	22 (73)		
< 1 year	1 (3)		
Unknown^c^	7 (23)		
10‐year atherosclerotic cardiovascular disease risk (ASCVD)			
ASCVD available, *n* (%)	23 (77)		
Median risk [IQR]	15 [7–20]		
Risk distribution, *n* (%)			
0 to < 7.5	6 (20)		
≥ 7.5 to < 20	11 (37)		
≥ 20	6 (20)		

Abbreviations: Diabetes, Type 2 diabetes mellitus; HIV, human immunodeficiency virus; IQR, interquartile range.

^a^ CD4 nadir imputed to 199 cells/mm^3^.

^b^ HIV viral load on day of study visit.

^c^ Individuals with a historical record outside of the institution.

The distribution of biological sex of participants was comparable between cohorts (rank sum pairwise comparisons all *p* > 0.05). The PWH cohort comprised an ethnically and racially representative population of those with HIV in Suffolk County, Massachusetts, including 40% Black or African American and 27% Hispanic participants. In comparison to PWH, the VC cohort comprised 32% Black (*p* > 0.05) and 16% Hispanic participants (*p* < 0.001). The MCI cohort comprised entirely White, non‐Hispanic participants (*p* < 0.005 compared to both PWH and VC cohorts). The cumulative presence of cardiovascular risk factors (namely hypertension, hyperlipidemia, atrial fibrillation, and type 2 diabetes mellitus) was higher in the PWH (*p* < 0.05) and VC (*p* < 0.001) cohorts compared to MCI, while PWH and VC cohorts were comparable (*p* > 0.05). MCI participants were older than those in the PWH and VC cohorts (both *p* < 0.001), while there was no statistically significant difference in age between PWH and VC participants (*p* > 0.05). Performance on the MoCA was comparable between all groups (all *p* < 0.05; see Table 1).

### 3.2 Volumetric Validation

**FIGURE 1:**
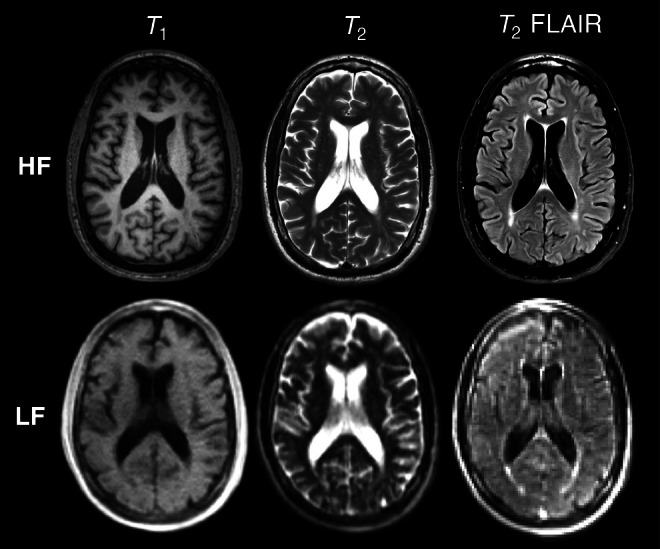
Comparison of high‐field (HF) and low‐field (LF) imaging acquired in a 63‐year‐old White Male with HIV. Representative example axial *T*_1_‐weighted, *T*_2_‐weighted and *T*_2_ fluid attenuated inversion recovery (FLAIR) images are shown for both HF and LF counterparts.

Available HF‐MRI scans acquired within 1 year of LF‐MRI were utilized as reference standards for validation (*n* = 9). Example axial *T*_1_‐weighted, *T*_2_‐weighted, and *T*_2_ FLAIR images acquired at HF compared with corresponding original axial LF scans are shown in Figure 1.

**FIGURE 2:**
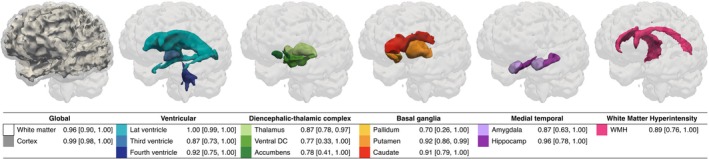
Correlation between low‐field (LF) derived segmentation volumes and high‐field (HF) counterparts. LF *T*_2_ FLAIR images were segmented using machine learning algorithm WHM‐SynthSeg in FreeSurfer and grouped into anatomical regions comprising global, ventricular, diencephalic‐thalamic, basal ganglia, medial temporal, and white matter hyperintensity volumes, as shown in 3D render. LF volumes were correlated with those derived from HF imaging counterparts and reported as Pearson's *r* and 95% confidence intervals [95% CI]. Accumbens, nucleus accumbens; Hippocamp, hippocampus; Lat ventricle, lateral ventricle; Ventral DC, ventral diencephalon, WMH, white matter hyperintensities. All *p <* 0.05.

Segmentation volumes derived from WMH‐SynthSeg were strongly correlated between LF and HF counterparts (Figure 2; Figure S1). Correlation coefficients of *r* > 0.96 were observed for the white matter and cortex (all *p* < 0.01). For the ventricular system, correlations *r* > 0.87 were observed (all *p* < 0.01). Correlations between DTC regions were *r* > 0.77 (all *p* < 0.05), basal ganglia regions were *r* > 0.70 (all *p* < 0.05), and medial temporal regions were *r* > 0.87 (all *p* < 0.01). Correlations were higher between larger structures (e.g., *r =* 1.0 for the lateral ventricles, *r* = 0.99 for the cortex), while smaller subcortical structures were lower (e.g., *r =* 0.70 for the pallidum, *r =* 0.78 for the nucleus accumbens). Globally, the ASPD was 10.89% for the white matter and 0.85% for the cortex, and spatial overlap between volumes as measured using the Dice coefficient was 0.78 and 0.73 respectively (Table S2). For ventricular structures, all ASPD were < 5% and Dice > 0.74. For the DTC regions, the ASPD was < 14% and Dice > 0.75; for the basal ganglia, ASPD was < 8% and Dice > 0.71; and for the medial temporal structures, the ASPD was < 6% and Dice was > 0.80.

WMH volumes were strongly correlated between LF and HF counterparts (*r* = 0.89, *p* < 0.01), while the ASPD was 13.45% and Dice was 0.48. Fazekas scores derived from LF images revealed a higher prevalence of WMH in the periventricular white matter (median 1, IQR 1‐1) relative to the deep white matter (median 0, IQR 0‐0), with a cumulative score of 1 (IQR 1‐2). ML derived LF WMH volumes were fairly correlated with the visually derived Fazekas scores in the periventricular white matter (*r* = 0.34, *p* = 0.04), deep white matter (*r* = 0.36, *p* > 0.05), and the cumulative score (*r* = 0.35, *p* = 0.04).

### 3.3 Association of ASCVD Risk With WMH Volume in PWH

The median 10‐Year ASCVD risk score in the PWH cohort was 15% (IQR 7–20), with 20% in the low/borderline risk category (0 to < 7.5%), 37% at intermediate risk (7.5 to < 20%), and 20% at high risk (≥ 20%) of the *n* = 23 PWH with available cholesterol labs within 18 months (median 3 months, IQR 0–14) of the study visit (Table 1). ASCVD risk was moderately correlated with WMH volume (*r* = 0.53 [95% CI 0.22, 0.84], *p* = 0.01), while correlations with cortical and subcortical volumes were fair (all *r* < 0.36 and *p* > 0.05). Post hoc analysis demonstrated that high risk ASCVD individuals had significantly greater WMH volume (14 cm^3^, IQR 12–15) compared to those at low/borderline risk (8 cm^3^, IQR 6–11; *p* < 0.01). A significant increase in WMH volume was also seen in those at intermediate risk (11 cm^3^, IQR 9–16) compared to those at low/borderline risk (*p <* 0.05). No differences in WMH volume were observed between high and intermediate risk individuals (*p* > 0.05).

### 3.4 Association of Cognitive Performance With Brain Volume in PWH

With respect to cognitive impairment, median performance on the MoCA was 23 (IQR 20–25) on a scale of moderate impairment [10–17], mild impairment [18–25], and normal cognition (≥ 26) of the *n* = 29 PWH with available MoCA scores (*n* = 1 not reported due to an incomplete score; Table 1). Lower performance on the MoCA was moderately correlated with smaller hippocampal (*r =* 0.40 [95% CI 0.05–0.75], *p* < 0.05) and cortical (*r* = 0.40 [95% CI 0.05–0.66], *p* < 0.05) volumes. Post hoc comparisons between non‐cognitively impaired (*n* = 6) and cognitively impaired groups (comprising mild (*n* = 20) and moderate (*n* = 3) impairment) demonstrated a trend toward a reduction in volume of the hippocampus (3.7 cm^3^, IQR 3.2–4.1 in impaired versus 3.9 cm^3^, IQR 3.6–4.2 in cognitively normal individuals) and a reduction in volume of the cortex (23 cm^3^, IQR 20–24 in cognitively impaired versus 25 cm^3^, IQR 22–27 in cognitively normal), although these were not significant (*p* > 0.05). No other associations were observed between MoCA and other cortical or subcortical volumes (all *r* < 0.36 and *p* > 0.05).

### 3.5 Multivariate Regression Analysis of Brain Volumes With HIV Characteristics in PWH

Multivariate regression analysis was performed to evaluate the association between brain volumes and HIV characteristics in those PWH when adjusted for age and sex. Volume of the white matter, caudate, and putamen did not reveal associations between HIV‐related characteristics including CD4+ T‐cell count, CD4+ T‐cell nadir, HIV‐1 viral load, and ART duration (all *p >* 0.05).

### 3.6 Patterns of Brain Volume Loss in PWH, Vascular, and MCI Cohorts

**FIGURE 3:**
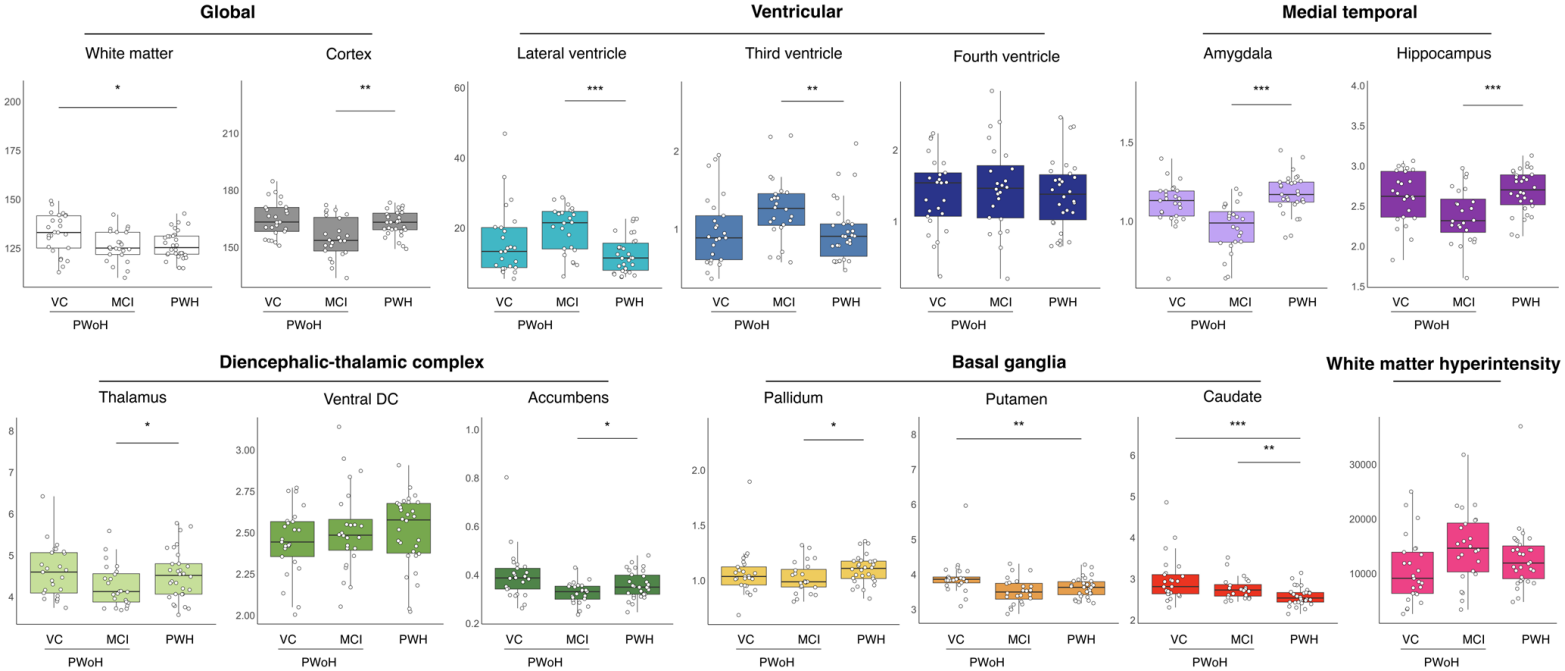
Patterns of atrophy in people with HIV (PWH) compared to people without HIV (PWoH) with vascular comorbidities (VC) or mild cognitive impairment (MCI). Patterns of atrophy in global, ventricular, medial temporal, diencephalic‐thalamic, basal ganglia, and white matter hyperintensity (WMH) regions. All regions were corrected for intracranial volume and are reported in mm^3^ × 10^3^, except for WMH, which is reported in mm^3^. Comparisons between groups were analyzed using Kruskal–Wallis with Dunn's post hoc tests. Data are presented as median, interquartile range, and individual data points. Accumbens, nucleus accumbens; Ventral DC, ventral diencephalon. *p < 0.05, **p < 0.01, ***p < 0.001.

LF segmentation volumes derived from PWH were compared to PWoH with at least one VC and probable vascular cognitive impairment (vascular comorbidities cohort; VC) or PWoH with MCI due to suspected AD (MCI). Analyses of global brain volume showed that PWH had reduced white matter compared to PWoH with VC (*p* = 0.01), a reduction which was also seen in those with MCI (Figure 3). Conversely, cortical volume and ventricular system volumes were similar between PWH and the VC, while the third and lateral ventricles were larger, and cortex was smaller in those with MCI compared to PWH (all *p* < 0.01). Subcortical volume analyses showed greater complexity: hippocampal, amygdala, and DTC subregion volumes, including the thalamus and nucleus accumbens, were significantly larger in PWH compared to individuals with MCI (*p* < 0.01 for hippocampal and amygdala volumes; *p* < 0.05 for the thalamus and accumbens). In contrast, putamen volumes were significantly smaller in PWH compared to the VC (*p* < 0.01), while caudate volumes showed significant reductions compared to both VC (*p* < 0.01) and MCI (*p* = 0.01) cohorts. Pallidum volume was higher in PWH compared to PWoH with MCI (*p* < 0.05). No significant differences were found in WMH volume among the groups (all *p* > 0.05).

### 3.7 Multivariate Regression Analysis of Brain Volumes in PWH, Vascular, and MCI Cohorts

Across cohorts, multivariate analysis was performed to evaluate associations between brain volumes in PWH compared to PWoH (VC and MCI) when adjusted for age and sex (Table 2). Reduced volume of the caudate, putamen, and white matter was independently associated with PWH relative to PWoH with VC (all *p* < 0.05). Reduced volume of the amygdala was independently associated with MCI relative to both PWH and PWoH with VC (all *p* < 0.05), while reduced cortical volume was observed in those with MCI relative to those with VC alone (*p* < 0.05). Volume of the hippocampus was comparable between cohorts, as was WMH volume (all *p* > 0.05).

**TABLE 2:** Multivariate linear regression outcomes. Multivariate linear regression analyses were performed to evaluate the relationship between volumes of interest and cohort when adjusted for age and sex. Pairwise comparisons between people with HIV (PWH) and people without HIV with vascular comorbidities (VC) or mild cognitive impairment (MCI) of adjusted marginal means with Bonferroni correction are reported as multiplicity‐adjusted *p* values, adjusted effect size, and 95% confidence intervals. Volumes were corrected for intracranial volume and are reported in mm^3^ × 10^3^ except for white matter hyperintensities (WMH), which is reported in mm^3^. PWH was the reference cohort in the model.

	*p* value	Effect size	95% CI lower limit	95% CI upper limit
Amygdala				
PWH versus MCI	0.001*	−0.17	−0.28	−0.06
PWH versus VC	0.628	−0.05	−0.14	0.04
VC versus MCI	0.016*	0.12	0.02	0.23
Caudate				
PWH versus MCI	0.091	0.26	−0.03	0.55
PWH versus VC	0.000*	0.41	0.16	0.66
VC versus MCI	0.578	0.15	−0.13	0.43
Cortex				
PWH versus MCI	0.331	−4.37	−10.98	2.25
PWH versus VC	0.695	2.82	−2.90	8.53
VC versus MCI	0.025*	7.18	0.69	13.67
Hippocampus				
PWH versus MCI	0.222	−0.17	−0.39	0.06
PWH versus VC	1.000	−0.04	−0.24	0.15
VC versus MCI	0.514	0.13	−0.10	0.35
Putamen				
PWH versus MCI	1.000	0.02	−0.27	0.32
PWH versus VC	0.012*	0.31	0.05	0.57
VC versus MCI	0.055	0.29	0.00	0.58
White matter				
PWH versus MCI	0.835	1.70	−3.47	9.06
PWH versus VC	0.019*	1.70	0.80	11.63
VC versus MCI	0.530	1.70	−2.72	9.57
WMH				
PWH versus MCI	1.000	−39.02	−4602.83	4524.79
PWH versus VC	0.396	−2452.45	−6395.21	1490.31
VC versus MCI	0.572	−2413.43	−6889.34	2062.49

* *p* < 0.05.

When considering the age and sex of PWH and PWoH, increasing age was independently associated with a reduced volume of the hippocampus, cortex, putamen, and white matter and increased WMH volume (all *p* < 0.05). No significant associations were observed for sex at birth; however, the number of women included in the PWH cohort was limited (all *p* > 0.05).

## 4 Discussion

In this study, we demonstrate the feasibility of integrating LF‐MRI with routine clinical evaluations in an outpatient clinic setting and the utility of ML algorithms in detecting regional atrophy in PWH. Brain volumes derived from LF‐MRI agreed with HF counterparts and demonstrated a distinct pattern of white matter and subcortical atrophy in PWH on ART. These findings highlight the potential of LF‐MRI to complement routine HIV care and enable longitudinal assessment of structural brain changes in PWH over the lifespan, with applications to other aging and at‐risk populations where traditional MRI access is limited.

Accelerated neuronal loss in PWH on ART is considered complex and multifaceted, involving factors such as HIV‐specific history (e.g., duration of infection, timing of ART initiation, history of immune suppression), behavioral considerations (e.g., smoking), or age‐associated vascular comorbidities (e.g., hypertension, diabetes, cardiovascular disease) that contribute to the overall risk profile for a reduction in brain health [8, 35, 36, 37, 38, 39]. The interplay of these diverse factors creates a unique pathophysiological environment that may increase the vulnerability of PWH to brain atrophy and cognitive disorders.

While HF‐MRI is often used in diagnostic procedures, PWH may experience disparities in access to neuroimaging due to disproportionate multimorbidity [40], poverty [41], stigma [42], access, and medical discrimination [43]. In this cross‐sectional LF‐MRI study performed in an outpatient clinic setting, specific regional atrophy in PWH was observed when compared with individuals of similar age without HIV. Notably, PWH had a reduction in white matter volume and subcortical atrophy in the putamen and caudate that was evident on LF‐MRI. This pattern was distinct from that of PWoH with a clinical diagnosis of MCI due to suspected AD, where atrophy was predominantly localized to the cortex and medial temporal lobe, including the hippocampus and amygdala. A key finding was the observed reduction in white matter, putamen, and caudate volume in PWH compared to PWoH with VC, irrespective of age. These findings highlight distinct patterns of brain volume loss in PWH, particularly affecting white matter and specific subcortical structures, which differ from patterns observed in individuals without HIV.

A substantial challenge for providers is differentiating brain injury due to HIV infection versus age‐associated neurodegenerative disorders. Discriminant atrophy in the basal ganglia is a hallmark of HIV [44, 45] with studies showing caudate atrophy persisting in PWH despite ART [46, 47]. Many participants in this study were treated during earlier eras before the implementation of the “test and treat” strategy [48]. Caudate volume loss, which in this study was associated with HIV diagnosis, is a finding consistent with other recent studies using HF‐MRI conducted in acute HIV cohorts and participants recruited for the AIDS Clinical Trials Group [46]. While attributing imaging findings to HIV alone is complex, this study demonstrates the unique capability of LF‐MRI to detect suspected HIV‐related brain changes, particularly in subcortical regions. When combined with readily accessible routine clinical evaluations, this highlights its potential as a valuable tool for monitoring brain health in PWH, especially in settings where HF‐MRI may not be readily available.

We observed that reductions in hippocampal volume were more strongly associated with increasing age rather than HIV diagnosis. This distinction suggests HIV‐specific effects and normal aging processes may be visualized on LF‐MRI. Interestingly, we did not observe any association between HIV infection duration and white matter, putamen, or caudate volume, nor did we observe any statistically significant associations with disease severity (CD4+ T‐cell count and CD4+ T‐cell nadir). However, it is important to note that our study may have been underpowered to detect such relationships given the sample size. While our cross‐sectional design precludes drawing conclusions about degeneration in these regions over time, prior research [49] suggests that atrophy in these regions may be mitigated by virally suppressive ART. Future longitudinal studies using LF‐MRI could offer valuable insights in tracking brain volumes in PWH engaged in care, potentially surpassing sample sizes typically achieved for HF‐MRI studies.

Although the PWH cohort in this study was racially and ethnically representative of the regional population, limitations include a small cross‐sectional analysis, limited sample size, use of historical cohorts for comparison, cognitive assessments limited to the MoCA, and the absence of an age‐matched cognitively normal cohort. Future studies in the field should focus on contemporaneous enrollment, comprehensive phenotyping of cognitive subtypes, assessment of factors that could modify dementia risk (e.g., substance use disorders), in addition to evaluating the accuracy of LF‐MRI in PWH longitudinally. Recruitment of individuals across multiple sites, particularly from those regions where HIV is most prevalent, access to care is limited, and resources are constrained, is also necessary to provide a more comprehensive, generalizable, and real‐world evaluation of the utility of LF‐MRI for the evaluation of PWH. These efforts should concomitantly seek to advance technical limitations associated with LF‐MRI acquisition and post‐processing, including strategies to enhance image resolution and downstream segmentation, particularly of subregions, which remains an area of active investigation.

In conclusion, our study demonstrates the utility of portable, LF‐MRI in accurately measuring brain volumes in PWH, with patterns observed comparable to those derived from HF‐MRI and frequently reported in the clinical literature. This highlights the potential of LF‐MRI technologies in advancing access to neuroimaging to facilitate early detection and monitoring of brain atrophy in individuals with HIV, with the potential to pair with cognitive assessment and blood biomarkers of neurodegeneration and improve long‐term health outcomes. To enable equitable and sustainable adoption in HIV care in areas with restricted access to HF‐MRI, future efforts must also focus on improving access, implementation, and interpretability of ML‐based neuroimaging. More broadly, this approach could also enhance care for underserved older adults and other populations at‐risk for dementia.

## Author Contributions

A.S.‐A., S.S.M., and W.T.K. contributed to the conception and design of the study; A.S.‐A., M.K., J.G., and D.D. contributed to the acquisition and analysis of data; A.S.‐A., M.K., J.G., D.D., R.A., K.Z., G.R., R.G., B.S., A.D.H., K.N.S., O.R., J.E.I.G., W.T.K., and S.S.M. contributed to drafting the text or preparing the figures.

## Conflicts of Interest

This study received research support from Hyperfine Inc. (W.T.K). Hyperfine had no role in the conceptualization, design, analysis, preparation of the manuscript, or decision to publish.

## Supporting information


**Data S1:** acn370237‐sup‐0001‐Supinfo.docx.

## Data Availability

The patient data are available to academic researchers under restricted access due to privacy and ethical restrictions, and access can be obtained by contacting the corresponding author and entering into an institutional data use agreement. WMH‐SynthSeg is publicly available and implemented in FreeSurfer: https://surfer.nmr.mgh.harvard.edu/fswiki/WMH‐SynthSeg.
