# Supplementary material for: Portable Low‐Field Magnetic Resonance Imaging in People With Human Immunodeficiency Virus
Source: Ann Clin Transl Neurol. 2025 Nov 6;13(3):537–46. doi: 10.1002/acn3.70237 (PMC12968453; doi:10.1002/acn3.70237)
Supplement: Supplementary file 1 — Data S1: acn370237‐sup‐0001‐Supinfo.docx. [file ACN3-13-537-s001.docx]

**SUPPLEMENTARY INFORMATION**

**Portable Low-Field Magnetic Resonance Imaging in People with Human Immunodeficiency Virus**

Annabel Sorby-Adams PhD^1,2*^, Malachi Keo BA^1*^, Jennifer Guo BS^1,2^, Daire Daly BA^1^, Richard Ahern NP^3^, Kimon Zachary MD^3^, Gregory Robbins MD^3^, Rajesh T. Gandhi MD^3^, Bragi Sveinsson PhD^4^, Adam de Havenon MD MS^5^, Kevin N. Sheth MD^5^, Otto Rapalino MD^6^, Juan Eugenio Iglesias Gonzales PhD^4,7,8^, W. Taylor Kimberly MD PhD^1,2†^, Shibani S. Mukerji MD PhD^1,9†^

^1^ Department of Neurology, Massachusetts General Hospital and Harvard Medical School, Boston, Massachusetts, US

^2^ Center for Genomic Medicine, Massachusetts General Hospital and Harvard Medical School, Boston, Massachusetts, US

^3^ Division of Infectious Diseases, Massachusetts General Hospital and Harvard Medical School, Boston, Massachusetts, US

^4^ Athinoula A. Martinos Center for Biomedical Imaging, Department of Radiology, Massachusetts General Hospital and Harvard Medical School, Boston, Massachusetts, US

^5^ Department of Neurology, Center for Brain & Mind Health, Yale New Haven Hospital and Yale School of Medicine, New Haven, Connecticut, US

^6^ Division of Neuroradiology, Department of Radiology, Massachusetts General Hospital and Harvard Medical School, Boston, MA, US

^7^ Centre for Medical Image Computing, University College London, London, UK

^8^ Computer Science and Artificial Intelligence Laboratory, Massachusetts Institute of Technology, Cambridge, Massachusetts, US

^9^ Vaccine and Immunotherapy Center, Massachusetts General Hospital, Boston, Massachusetts, US

* ^†^ Authors contributed equally

Correspondence: Shibani Mukerji

Corresponding author:

Shibani Mukerji, MD, PhD

Massachusetts General Hospital

Harvard Medical School

114 13th St 2nd Floor JJ,
Charlestown, MA 02129-2020
857-282-9950

smukerji@mgb.org

**Supplementary Table 1.** Image acquisition parameters

|  | ***T*_1_** | ***T*_2_** | ***T*_2_ FLAIR** | ***T*_1_** | ***T*_2_** | ***T*_2_ FLAIR** |
| --- | --- | --- | --- | --- | --- | --- |
| TE | 5.41 | 216.4 | 169.2 | 4.36 | 169.2 | 152.3 |
| TR | 880 | 2000 | 3500 | 880 | 2000 | 3500 |
| TI | 334.5 | – | 1296 | 351.5 | – | 1308 |
| Plane | Axial | Axial | Axial | Isotropic | Isotropic | Isotropic |
| In-plane resolution (mm) | 1.6 × 1.6 | 1.6 × 1.6 | 2 × 2 | 3.0 × 3.0 | 3.0 × 3.0 | 3.0 × 3.0 |
| Slice thickness (mm) | 5 | 5 | 5.88 | 3 | 3 | 3 |
| Flip angle (°) | 90 | 90 | 90 | 90 | 90 | 90 |
| Acquisition length (mm:ss) | 4:04 | 3:00 | 5:49 | 3:32 | 2:30 | 8:05 |

FLAIR – fluid attenuated inversion recovery, TE – echo time, TI – inversion time, TR – repetition time.

**Supplementary Figure 1.** Correlations of brain volumes derived from low-field (LF) MRI with high-field (HF) counterparts. Correlations between LF and HF-MRI counterparts are reported as Pearson’s *r*, *p*-value, and 95% confidence intervals from global, ventricular, medial temporal, diencephalic-thalamic and basal ganglia regions of interest, and white matter hyperintensities. Accumbens – nucleus accumbens; Ventral DC – ventral diencephalon.

**Supplementary Table 2.** Dice coefficient and absolute symmetrized percent difference (ASPD) reported as median and 95% confidence intervals (CI).

|  | **ASPD** | **Dice** |
| --- | --- | --- |
|  | **Median [95% CI]** | **Median [95% CI]** |
| Global |  |  |
| White matter | 10.89 [7.60, 14.18] | 0.78 [0.78, 0.79] |
| Cortex | 0.85 [0.13, 1.83] | 0.73 [0.71, 0.74] |
| Ventricular | |  |
| Lateral ventricle | 4.62 [2.44, 6.81] | 0.88 [0.83, 0.94] |
| Third ventricle | 3.10 [0.82, 7.03] | 0.79 [0.76, 0.83] |
| Fourth ventricle | 3.85 [2.60, 5.11] | 0.74 [0.70, 0.78] |
| Diencephalic-thalamic complex | | |
| Thalamus | 13.71 [7.51, 19.91] | 0.85 [0.83, 0.87] |
| Ventral DC | 7.89 [1.18, 14.61] | 0.78 [0.76, 0.81] |
| Accumbens | 7.89 [0.98, 14.80] | 0.75 [0.69, 0.81] |
| Basal ganglia | |  |
| Pallidum | 7.44 [1.93, 16.82] | 0.71 [0.65, 0.76] |
| Putamen | 3.82 [0.24, 7.88] | 0.83 [0.82, 0.84] |
| Caudate | 5.53 [0.21, 10.84] | 0.84 [0.83, 0.86] |
| Medial temporal | |  |
| Amygdala | 5.62 [1.37, 9.87] | 0.80 [0.78, 0.82] |
| Hippocampus | 2.85 [0.36, 6.06] | 0.80 [0.77, 0.82] |
| White matter hyperintensity | 13.45 [8.00, 18.89] | 0.48 [0.29, 0.66] |

Ventral DC – ventral diencephalon.
